# Characterizing the linguistic profiles, training needs, and caseloads of speech language pathologists providing clinical services to multilingual people with aphasia: The international Multilingual Aphasia Practices (MAP) consensus group survey

**DOI:** 10.1371/journal.pone.0346488

**Published:** 2026-04-09

**Authors:** Seçkin Arslan, Monica I. Norvik, Reem S. W. Alyahya, Javad Anjum, Valantis Fyndanis, Ritienne Grima, Silvia Martínez-Ferreiro, Amaia Munarriz-Ibarrola, Claudia Peñaloza, John E. Pierce, Marie Pourquié, Grégoire Python, Suzan D. Scheffer, Eva Soroli, Wei Ping Sze, Maria Kambanaros, On behalf of the Multilingual Aphasia Practices consensus group (MAP, The Collaboration of Aphasia Trialists Working Group 2)

**Affiliations:** 1 CNRS, BCL, Université Côte d’Azur, Nice, France; 2 Department of Education, Faculty of Humanities, Social Sciences and Education, UiT The Arctic University of Norway, Tromsø, Norway; 3 Department of Allied Health, School of Health and Medical Sciences, City St George’s, University of London, London, United Kingdom & King Fahad Medical City, Saudi Arabia; 4 Department of Communication Sciences and Special Education, University of Georgia, Athens, Georgia, United States of America; 5 Department of Rehabilitation Sciences, Cyprus University of Technology, Limassol, Cyprus; 6 Department of Human Communication Sciences & Disorders, University of Malta, Msida, Malta; 7 Gerontology and Geriatrics Research Group, Instituto de Investigación Biomédica de A Coruña (INIBIC), Complexo Hospitalario Universitario de A Coruña (CHUAC), SERGAS, Universidade da Coruña, A Coruña, Spain; 8 ELEBILAB, University of the Basque Country UPV/EHU, Vitoria-Gasteiz, Spain; 9 Department of Cognition, Development and Educational Psychology, Faculty of Psychology, University of Barcelona, Barcelona, Spain; 10 Centre of Research Excellence in Aphasia Recovery and Rehabilitation, La Trobe University, Melbourne, Australia; 11 Faculty of Psychology, University of Geneva, Geneva, Switzerland; 12 Department of Artificial Intelligence, University of Groningen, Groningen, The Netherlands; 13 Department of Linguistics, University of Lille, CNRS, UMR - STL - Savoirs Textes Langage, Lille, France; 14 Division of Psychology and Language Sciences, University College London, London, United Kingdom; Manipal Academy of Higher Education, INDIA

## Abstract

Globally, there is still limited understanding of how Speech-Language Pathologists (SLPs) assess and treat multilingual people with aphasia (MPWA). This article presents results from the Multilingual Aphasia Practices (MAP) survey—an extensive international study involving 407 SLPs working across 60 countries. The MAP survey explored: 1) the multilingual background of SLPs and the languages they incorporate into service delivery, 2) their knowledge and professional training related to multilingualism and multilingual aphasia, and 3) their workplace contexts and client profiles. A large proportion of respondents (79.7%) identified as multilingual and reported using numerous languages in their practice. However, formal training in multilingualism was often minimal. Only 25.06% had completed a course focused on multilingualism, and just 10.07% took a full course specific to multilingual aphasia. Most participants (87.2%) reported major gaps in knowledge and training, particularly regarding best-practice recommendations, supervised clinical experience, and guidance on assessment and intervention for MPWA. Many expressed a strong desire for additional professional development in these areas. Clinical exposure to MPWA varied widely. While 27% of respondents reported daily contact, 26.1% encountered MPWA once or twice per week, and 22.8% indicated that they worked with MPWA only a few days per month. Overall, our findings point to persistent and widespread global gaps in training, resources, and clinical readiness for working with MPWA. The results underscore an urgent need to enhance multilingualism-focused education in SLP programs, establish international best-practice frameworks, develop and disseminate culturally and linguistically appropriate assessment and treatment materials.

## 1. Introduction

More than half of the global population speaks more than one language on a daily basis, reflecting the fundamentally multilingual nature of our world. Nonetheless, multilingualism varies widely depending on cultural, regional, and national contexts [1]. We use the term ‘multilingual’ to refer to any individual who speaks more than one language in their daily lives [2]. In this article, we investigate current practices of Speech and Language Pathologists (SLPs) working with multilingual people with aphasia (MPWA) around the world. Aphasia is an acquired neurogenic language/communication disorder resulting from neural damage to the brain’s language processing network and is characterized by impairments in language comprehension and/or production across language modalities. Aphasia affects individuals, their families, and their broader communities by restricting communication, reducing independence in daily activities, limiting social participation, and hindering employment opportunities. These impairments also impose a tremendous public health burden, with estimates of about 12 million people living with aphasia across the globe [3]. Due to expanding numbers of multilingual individuals, multiethnic and multilingual aphasia caseloads are also rapidly growing in clinical practice [4,5]. The variable nature of multilingualism, shaped by a multitude of factors including language contact, typological diversity, sociopolitical upheavals, and migration, adds a significant level of complexity and challenge to SLPs’ practices when delivering clinical services for MPWA. These challenges include linguistic diversity of MPWA [6–8] and strategies to address this diversity, such as the use of trained interpreters during service delivery (see, e.g., [9]). To improve services and outcomes for MPWA, multilingualism warrants focused scholarly and clinical attention [4,10–14].

The current understanding of SLP practices in delivering clinical services for MPWA remains fragmented and is primarily informed by surveys conducted within specific countries and regions, and as a result, a comprehensive global perspective has so far not been achieved. Below, we outline the principal contributors and geographic regions in which multilingual aphasia practice research has been most prominently represented.

### 1.1. North America — Centeno [15,16] and D’Souza [17]

Centeno [15] noted that MPWA, especially those belonging to minority language conditions such as Spanish-speaking Hispanic individuals in the U.S., frequently face reduced access to clinical SLP services, and that SLPs providing clinical services to MPWA encounter barriers including a lack of valid assessment tools and limited interpreter services. Centeno [16] later conducted a survey using a 36-item questionnaire that covered six areas: work setting and caseload, professional training, tools and procedures, service delivery practices, clinical challenges, and suggestions for improvement. The survey was completed by 125 SLPs in the U.S. A large majority of Centeno’s [16] respondents, around 85%, reported that they worked with MPWA, while only one-fifth of them identified as multilingual. Nevertheless, around 77% of respondents reported receiving little or no training to prepare them to provide clinical services to MPWA. Most SLPs reported that they use professional interpreters, bilingual colleagues, or family members to bridge linguistic gaps.

D'Souza [17] surveyed responses from 384 SLPs across Canada, gathering data on their demographics, languages spoken, caseloads, clinical practices, and perceived barriers. Although this survey did not specifically target SLPs who worked with MPWA, the author reported important findings regarding multilingual aphasia practices in Canada. D'Souza [17] found that the SLP respondents in Canada spoke 32 different languages, English and French being the most frequently spoken. Similar to Centeno [16], nearly 80% of respondents reported a lack of available aphasia assessment tools in languages other than English/French, and approximately 77% reported a shortage of professional SLPs speaking the client’s language.

### 1.2. Europe — Norvik et al. [18] and Pourquié [19]

Based on a survey conducted in Norway, Norvik et al. [18] reported findings in the European context similar to those in North America. The authors analyzed responses from 53 Norwegian SLPs, who reported working with MPWA in their everyday practice. A large proportion of Norvik et al.’s [18] respondents (81%) reported having received no or minimal training during professional training that would prepare them for working with MPWA. While the most commonly assessed language appeared to be Norwegian, slightly more than half of the respondents reported that other languages spoken by MPWA living in Norway were often not assessed. The most frequently reported challenges faced by SLPs included the lack of treatment (77%) and assessment tools (74%), as well as the lack of SLPs’ competence in MPWA’s first or the most proficient language other than Norwegian (68%).

Pourquié [19] surveyed 421 SLPs who speak French (n = 202), Spanish (n = 119), English (n = 80), Basque (n = 12), Portuguese (n = 6), and Russian (n = 1). The author showed that SLPs believed that multilingualism is highly relevant to the field of SLPs and that many of them worked in a multilingual context on a daily basis (with only 1% reported having no bilingual clients at all). The SLPs reported a lack of materials appropriate for multilingual contexts in which they work. Pourquié [19] further showed that the majority of SLPs who spoke French, Spanish, or Basque (but not those who spoke English) had not received any specific training on multilingualism while the vast majority felt that it was necessary.

### 1.3. India — Niharika et al. [20] and Chandana et al. [21]

Niharika et al. [20] conducted a recent survey with 75 SLPs practicing in clinical settings across India. Most respondents reported belonging to southern Indian ethnolinguistic communities, speaking Kannada or Malayalam as their first languages, and English and Hindi as their second languages. This survey study was not exclusively directed at SLPs working with MPWA; most respondents reported working with both children and adult caseloads. Nonetheless, results showed a similar finding to surveys in North American and European contexts. Niharika et al. [20] found that more than half of the respondents use interpreters during clinical service delivery and reported the lack of normative data and substantial dialectal variation as key barriers. Finally, almost all participating SLPs (93%) expressed that they lacked adequate training for delivering services to multilingual clients.

Using a survey administered to SLPs in India working with MPWA, Chandana and colleagues [21] recorded responses from 205 respondents. These respondents indicated that many Western assessment tools in use lack cultural appropriateness for the Indian context, potentially leading to MPWA not fully understanding test stimuli. Around 80% of respondents identified regional, dialectal and cross-linguistic differences to be the most significant barriers, followed by insufficient training.

### 1.4. South-East Asia — Guo et al. [22] and Hassan et al. [23]

Emerging work from Southeast Asian countries suggests that challenges and barriers faced by SLPs may be similar. For instance, Guo and colleagues [22] surveyed 36 clinicians providing SLP services for aphasia in Singapore and found that cultural and linguistic diversity were perceived as amongst the most significant challenges. Additionally, Hassan et al. [23] analyzed responses from 54 SLPs working with MPWA in Malaysia, representing different ethnicities, including Malay, Chinese, or Indian. They may therefore differ in their language backgrounds. At least 60% of respondents reported in Hassan et al. [23], indicated that they always have difficulty communicating with MPWA in their first or most comfortable language. Unsurprisingly, the most commonly reported challenges included an inability to understand and/or speak the client’s first language(s), followed by the lack of linguistically appropriate tools, and a shortage of SLPs competent in other languages or dialects spoken in Malaysia.

### 1.5. Oceania and the Pacific — Jodache et al. [24], Siyambalapitiya et al. [25] and Roger et al. [26]

Similar challenges and barriers have been documented in Oceania and the Pacific, including Samoan-speaking populations in New Zealand [24], and linguistically diverse communities in Australia [25]. For instance, Roger and colleagues’ [26] survey which involved 40 SLPs in the Sydney area, identified 39 different languages spoken by clients with aphasia. Respondents indicated that assessing aphasia through an interpreter and the lack of appropriate assessment tools as the most common barriers to providing effective clinical services.

### 1.6. Summary of survey studies so far

Taken together, previous surveys present a consistent picture of the challenges faced by SLPs when delivering clinical services to MPWA: respondents report limited or no formal training in multilingual/multicultural service delivery, a lack of linguistically and culturally appropriate assessment and/or treatment tools, a lack of competence in their clients’ first language and limitations when working with interpreters during service delivery, a practice reported across different studies. Yet, bringing these findings together, Ardila and Ramos’ [1] statement that multilingualism across the globe is highly variable, seems to be well reflected in the data across these survey studies. That is, while SLPs across different countries and regions reported similar challenges and barriers, the specific nature of these challenges could differ across contexts. For example, in Canada and Australia, challenges predominantly stem from the large number of languages spoken by indigenous or immigration-related minority language communities, whereas challenges in India, Singapore or Malaysia are sometimes related to a wide range of spoken dialects. There are clear gaps in aphasia practices worldwide, including a lack of adequate aphasia assessment tools and treatment materials [27,28] and a persistent Anglocentricity in available research and assessment tools that works against linguistic diversity [29,30]. Despite promising efforts made by different countries and regions, a comprehensive global understanding of clinical practices involving MPWA has yet to materialize. This is the central topic of the current study. Our international collaboration conducted a global survey to assess SLPs’ needs and challenges in providing clinical services to MPWA and the current state of multilingual aphasia care worldwide.

#### 1.6.1. The current survey study.

The overarching aim of this ‘MAP consensus’ study is to identify global practices in delivering clinical services to MPWA from the perspectives of SLPs. To this end, we developed a 32-item survey conducted worldwide. In part inspired by Centeno [16], the present study addresses the following research questions:

1. To what extent are SLPs multilingual, and how frequently do they use multiple languages when providing clinical services to MPWA?
2. What theoretical knowledge and clinical experience do SLPs possess when working with MPWA?
3. What are SLPs’ typical clinical settings and caseload characteristics of MPWA?

Following previous country-specific survey findings (see, e.g., [18,21,23]), we predicted that SLPs across the globe would underscore the lack of available training opportunities and clinical resources, as well as the challenges of working with MPWA from diverse multilingual and multicultural backgrounds. However, we also anticipated important differences depending on respondents’ demographic profiles, country of training or profession, and language backgrounds. Whether SLPs are multilingual or not, and the nature of the sociolinguistic status of the languages they speak, may lead to differences, including potentially subtle variations, within this international dataset that is unprecedented in scale. In this study, we report data from the following three sections in the survey: (I) demographic information, (II) educational background and training, and (III) clinical services until and including Q3.4 (see below). Survey findings concerning assessment and treatment practices, including SLPs’ suggestions, perceived barriers, and the different tools they use, will be reported in greater detail in Norvik et al. [31].

## 2. Methods

This survey is part of a larger consortium initiative conducted by the Multilingualism Aphasia Practices Consensus Group (MAP), assembled under Working Group 2: Aphasia Assessment and Outcomes of the Collaboration of Aphasia Trialists https://www.aphasiatrials.org/aphasia-assessments/. In the current article, we report on a part of the MAP survey, which was administered to a total of 407 SLPs worldwide, examining conceptual knowledge and clinical training, work settings and caseloads. The full survey is available in the Supplementary Materials. Anonymized data and codes, materials, and earlier preprints of this survey study can be found online on the OSF platform: https://osf.io/uzvn6/.

The project was approved by the ethics committee of the University of Groningen [CETO File no: 83938117]. Respondents received information on the survey’s purpose and were asked to provide explicit consent to participate. Our reporting of methods and results align with the Checklist for Reporting Results of Internet E-Surveys CHERRIES [32].

### 2.1. Survey design

The survey was first designed in English by the MAP consensus group. The group was first assembled in April 2020 and comprises 18 experts representing 13 countries. All questions were extensively discussed and reviewed between February 2021 and April 2022, with agreement reached by group consensus that considered the different multilingualism situations across countries for inclusivity.

The final version of the survey included both closed quantitative “core” items and open-ended qualitative items for “follow-up” or “clarification” purposes. Overall, there were 32 questions organized in four sections: 1) SLPs’ demographic information (8 questions); 2) Educational background and training related to multilingualism and MPWA (9 questions); 3) Clinical services provided to MPWA (11 questions); 4) Clinical assessment tools and therapy considerations for MPWA (4 questions). The full survey is available in the Supplementary Materials and on the OSF platform (https://osf.io/uzvn6/).

After the survey was developed, an external aphasia expert, JC, evaluated all questions to ensure clarity. He identified certain issues with 8 of the 32 questions devised in the MAP survey (questions 1.7, 2.4, 2.5, 2.6, 2.7, 3.9, 3.10, and 4.3). To ensure the transparency and quality of the survey, seven experts in aphasia (VF, SO, RG, GP, CL, SDTS, MT), who were not directly involved in the survey development, assessed the eight flagged questions using five steps of the *Question Appraisal System* (QAS-99; [33]): Instructions, Clarity, Assumptions, Response Categories, and Other. This resulted in a total of 17 QAS questions per MAP question (see supplementary materials on the OSF platform). The QAS questionnaire respondents saw multiple survey items per webpage. The questionnaire was distributed over 13 pages. Survey items were not randomized as all domains needed to be presented in a logical order due to the nature of the constructs measured. Based on their feedback, the questions were revised and re-evaluated by the MAP group, resulting in the final version of the survey.

### 2.2. Participants, administration, and analyses

Convenience sampling was used for this survey. Following Cochran [34], we estimated that a sample size of 385 individuals was required to achieve responses at a 95% confidence level. In order to meet the required estimated sample size, the MAP survey was first advertised within the CATs network in September 2022 and distributed to SLPs. The survey was distributed globally, initially in 41 countries, and later spread to over 60 countries across Europe, Asia, Africa, North America, and South America. The dissemination relied on more than 100 individual contacts, professional associations, research groups, and institutional networks, including national SLP associations, universities, hospitals, and international organizations (e.g., ESLA, IALP, ASHA communities). These contacts were asked to further circulate the survey within their professional networks, substantially expanding the potential reach of the study (the full list of networks can be found under survey distribution strategies on the OSF platform (https://osf.io/uzvn6/).

These recruitment efforts returned a total of 407 survey respondents who consented to participate, ensuring a larger sample size than initially estimated. Survey participation was anonymous and no personally identifiable information was analyzed (i.e., ages, names, phone numbers, IP numbers, or other electronic identifiers). Anonymized survey data without any sensitive personal identifiers was stored electronically in line with the General Data Protection Regulation (GDPR).

Before participants began responding to survey questions, we obtained their explicit written consent to participate voluntarily in this survey study, while confirming that the respondent: (a) was a professional SLP, (b) provided clinical services to people with aphasia, and (c) was older than 18. No monetary remuneration was offered.

The recruitment flyers and the survey were translated into 14 languages, including Arabic, Basque, Croatian, Dutch, French, Galician, German, Greek, Italian, Maltese, Mandarin Chinese, Norwegian, Spanish, and Turkish. Survey respondents were instructed to choose and respond in the language in which they felt most comfortable. Out of a total of 407 responses, English was the most frequent language that the respondents selected (n = 219, 53.8%), followed by French (n = 45, 11.1%) and Turkish (n = 30, 7.4%). Other languages included Spanish (n = 26, 6.4%), German (n = 24, 5.9%), Greek (n = 15, 3.7%), Italian (n = 13, 3.2%), Norwegian (n = 12, 2.9%), Dutch (= 8, 2.0%), Croatian (n = 6, 1.5%), Basque (n = 4, 1.0%), Galician (n = 2, 0.5%), Simplified Mandarin Chinese (n = 2, 0.5%), and Arabic (n = 1, 0.2%).

Data were collected and stored online via Qualtrics (Provo, UT). While responding to the survey, the participants were allowed to go back and review their responses before submission. Upon completion of the survey, each respondent was automatically given a unique respondent number. The respondents were instructed to keep this number safe in case they wished to alter or remove their responses later. We did not calculate view rate (i.e., viewers divided by unique responders) nor conduct an IP check, as such personal sensitive data were not kept in order to maintain anonymity. Data collection was conducted between April 2023 and February 2024. The data were accessed in CSV format, and responses were checked for completeness. Descriptive statistical analyses were computed in R using the *tidyr* and *dplyr* packages [35,36].

A total of five questions under the currently presented survey sections contained open-ended questions. The participants responded to these questions in the language that they were most comfortable with, and the responses were then translated into English by the members of our MAP group. At least 25% of the open-ended responses were coded independently by two experts. Agreement levels were high across all coded items: For 2.3_4 *Training in disciplines other than SLP?* agreement reached 85.19% (46/54 out of 151 responses), for 2.5 *Content of courses on bi/multilingualism and/or bi/multilingual aphasia* agreement was 87.30% (110/126 out of 207 responses). For both 2.9_6 *What is missing in your educational/professional training?* and 3.1_5 *How often do you work with people with aphasia?* coding interrater reliability was 100% (15/15 and 5/5 of all responses respectively). Finally for 3.3_5 *How often do you work with bi/multilingual people with aphasia?* agreement reached 95% (19/20 out of 68 responses). Overall, the high levels of agreement indicate strong reliability in the coding of open-ended responses.

Summarizing, the current paper reports responses to the following survey questions: *(I) Demographic information:* Q1.1–Q1.5 age, gender, employment, years of experience, multilingualism status; Q1.6 country of practice; Q1.7- Q1.8 languages or dialects spoken in general and used in clinical care. *(II) Educational background and training*: Q2.1–Q2.4 educational level, country of training, additional training, training in multilingualism; Q2.5 training content in multilingualism and multilingual aphasia; Q2.6 preparedness for working with MPWA; Q2.7 satisfaction with training; Q2.8 perceived importance of training; Q2.9 gaps in training. *(III) Clinical services:* Q3.1–Q3.2 frequency of working with PWA proportion of aphasia cases in the overall caseload; Q3.3–Q3.4 frequency of working with MPWA, proportion of multilingual aphasia cases in the overall caseload.

## 3. Results

### 3.1. Respondents’ demographic and linguistic backgrounds

**Fig 1:**
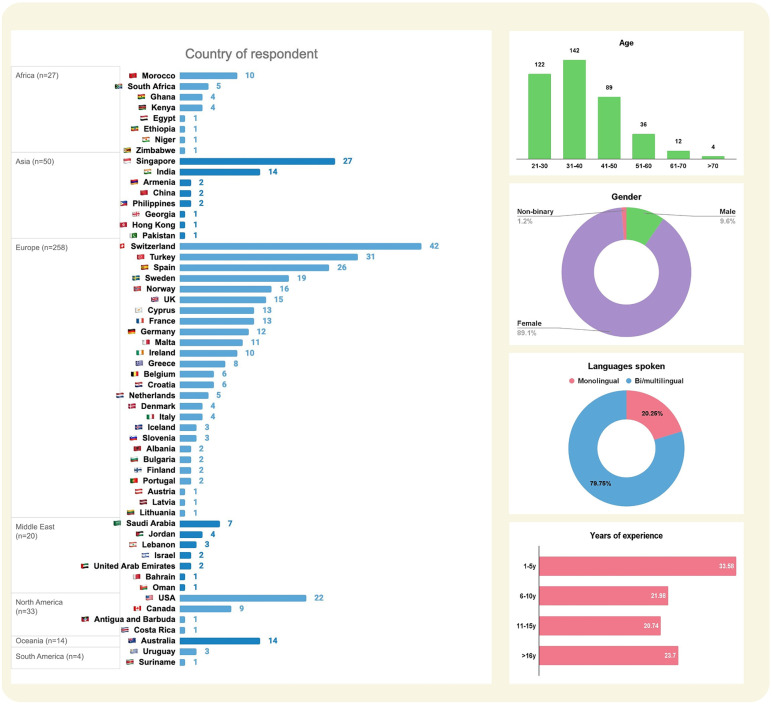
Respondents’ background information. The left panel indicates the global distribution of respondents’ country of practice. The right panel from top to bottom shows respondents’ distribution across age groups, gender values, bilingual status (monolinguals vs bi/multilinguals), and clinical experience levels.

A total of 407 respondents completed the survey. Fig 1 demonstrates a summary of demographic details, including the respondents’ country of practice, age, gender, and years of experience as an SLP. The respondents reported 60 different countries of practice and 55 different countries where they were trained (see Appendix 1 for details). Their ages ranged from 21 to over 70 years: 21–30 (n = 122), 31–40 (n = 143), 41–50 (n = 89), 51–60 (n = 37), 61–70 (n = 12), and >70 (n = 4). Most respondents were female (n = 362, 88.9%), while 40 identified as male (9.8%) and 5 identified as non-binary or preferred not to specify their gender.

The majority of respondents were employed full-time to provide clinical services (n = 276, 67.81%), while a small proportion worked part-time (n = 92, 22.60%). Fewer respondents identified themselves as unemployed (n = 9, 2.21%), retired (n = 5, 1.23%) or under the category ‘other’ (n = 12, 2.9%). About one-third of our respondents had 1–5 years of clinical experience (n = 136, 33.42%), followed by those with 6–10 years (n = 89, 21.87%), 11–15 years of experience (n = 85, 20.88%) and more than 16 years of experience (n = 97, 23.83%). More than half of them reported holding a postgraduate/master’s degree (n = 267, 70.45%, including 51 participants with a PhD), and around one-third held a bachelor’s degree (n = 112, 29.55%).

Out of 407 respondents, 323 (79.36%) reported being bi/multilingual speakers of at least more than one language in addition to their first acquired language. The SLPs were further required to list the languages they spoke, and we received responses from 324 respondents reporting a total number of 618 responses (i.e., they reported multiple languages). About a quarter of respondents listed English as one of their languages (n = 148, 23.9%), followed by French (n = 54, 8.7%), German (n = 41, 6.63%), Spanish (n = 33, 5.34%), Mandarin Chinese (n = 21, 3.40%), Italian (n = 19, 3.07%), Hindi (n = 12, 1.94%), and Turkish (n = 9, 1.46%). All other languages were reported by 288 participants (46.60%).

Respondents were also asked to rank their languages or dialects with which they communicate with MPWA while delivering clinical services, in the order of frequency of use within the last five years. A total of 108 unique languages/dialects were reported. English was the most often used language among the respondents, which, however, also emerged as the second most frequently used L2 of SLPs (45.1%) than as the most frequently used L1 (27.46%). Other most frequently spoken L1s included French (9.07%), Turkish (8.06%), Spanish (5.54%), Swedish (5.03%), Arabic (4.78%), and Norwegian (4.53%); and other most frequently spoken L2s included Spanish (8.69%), French (6.94%), Mandarin Chinese (6.6%), German (5.21%), Basque (3.47%), and Russian (2.08%) (for an exhaustive list of languages spoken, please see Appendix 2). We instructed the respondents to rate their self-perceived proficiency in the languages they use in clinical practice to provide clinical services to MPWA on a 5-point scale (1 = Very low; 5 = Very high). Respondents reported having highly proficient in their L1 (4.77, *SD* = 0.57), followed by L2 proficiency (3.69, *SD* = 1.20), L3 (2.90, *SD* = 1.12), L4 (2.36, SD = 1.14), and L5/Other (1.95, *SD* = 1.19).

### 3.2. Conceptual knowledge and training

Sections Q2.1 to Q2.5 enquired what best described the participants’ educational training. Most participants held a master’s degree (n = 216, 56.99%), followed by those with a bachelor’s degree (n = 112, 29.55%) and those with a PhD degree (n = 51, 13.46%). Many of these respondents received training solely in SLP (n = 219, 57.8%), while other respondents indicated receiving training in disciplines other than SLP (n = 160, 42.21%). A total of 379 participants out of 407 responded to the question regarding the highest level of degree obtained (Q2.1). Among participants who reported receiving training outside of SLP, their backgrounds included Languages, Literature, and Linguistics (23%), Health, Medicine, Therapy and Rehabilitation (20%), Psychology and Sociology (18%), and Education (14%). Less frequent responses were distributed across the following categories: Business, Administration, and Law (7.62%), Natural Sciences (5.24%), Arts and Humanities (except Languages) (4.76%), Information and Communication Technologies (2.86%), Null response, unspecified, field unknown (2.38%), and Welfare (1.43%).

**Fig 2:**
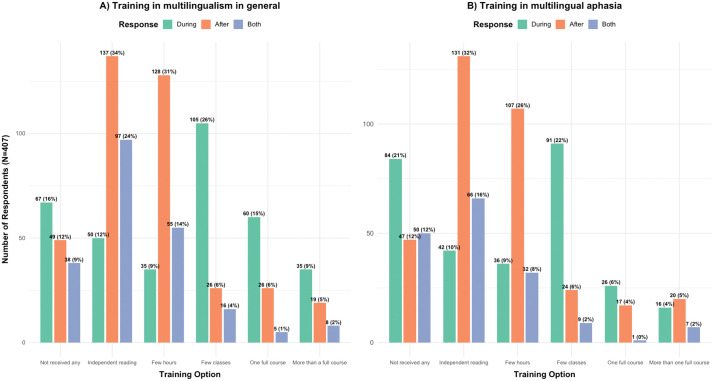
Q2.4 and Q2.5: *SLPs’ formal academic or clinical training received on A) multilingualism in general and B) multilingual aphasia.* Bar colors show the kinds of training respondents received before, after or both before and after their graduation as an SLP. Numbers of respondents per category are provided, percentages are rounded and based on a total of 407 respondents.

Questions Q2.4 and Q2.5 asked what best described their formal academic or clinical training received on multilingualism in general and in multilingual aphasia in particular, respectively, and whether they received such training opportunities before or after their graduation as an SLP (Fig 2). Full data tables summarizing response outputs from Likert questions regarding professional training Q2.4 to Q2.7 can be found in Tables A4_1, A4_2, A4_3, and A4_4 under Appendix 4.

Fig 2 presents the frequency and percentage of responses to items regarding the SLPs’ training received on multilingualism. Strikingly, the data revealed that a proportion of respondents either did not receive any training on multilingualism in general (16.4%) or, more specifically, on multilingual aphasia (20.6%), or they only received a few hours of lectures during their studies (31.4% on multilingualism theory and 26.2% specifically on multilingual aphasia). Additionally, 33.6% and 32.1% of respondents sought to address this gap in their training on multilingualism and multilingual aphasia, respectively, by independently reading on these topics after their studies. Those who received one or more full courses on these topics were very few (around 12% or less of the total respondents).

The survey was designed in a way that if the respondents clicked any of the options that indicated some level of training, an optional question (Q2.5.1) required them to provide the content of the courses or training they received. A total of 247 participants provided a response for this question, including multiple-word responses containing information that deserved more than one code (65 responses were selected). Most popular responses included language assessment and diagnostics (29.41%), followed by aphasia rehabilitation and recovery (27.23%) and general linguistics, psycholinguistics, and neurolinguistics (11.33%). The complete set of results is found in Appendix 3.

**Fig 3:**
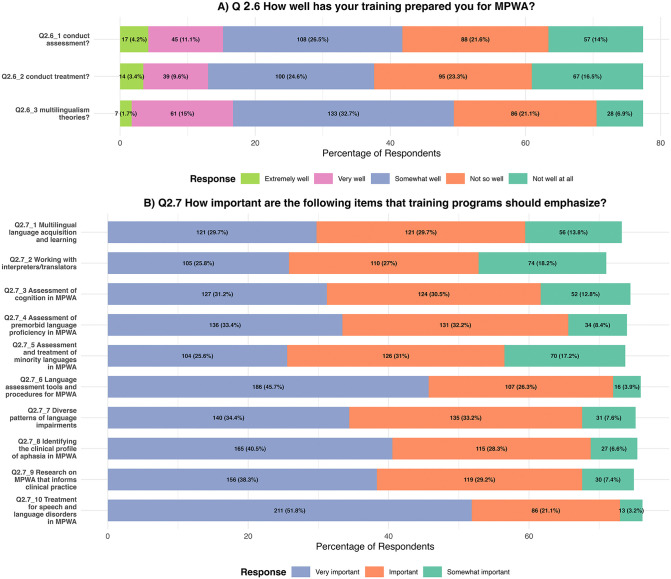
Q2.6 & Q2.7: *SLPs’ responses regarding A) how well they believe that their formal training prepared them for providing clinical services for MPWA and B) whether they believe professional clinical training should emphasize the contents listed.* In Fig 3B, the response options ‘not important’ and ‘not important at all’ are not depicted due to the very low number of responses. Percentages are rounded.

Fig 3 presents the frequency and percentage of respondents for questions Q2.6 and Q2.7, which asked participants to evaluate how adequately their training prepared them to provide clinical services for MPWA, and to indicate whether professional clinical education should place greater emphasis on elements they felt were missing.

As shown in Fig 3A, the most common response to all three questions inquiring how well the respondents were professionally trained to provide clinical services to MPWA was ‘somewhat well’. Specifically, among the 407 respondents, 108 (26.5%) selected this option for the assessment question, 100 (24.5%) for the treatment question, and 133 (32.7%) for the question addressing multilingualism theory. Fewer than 11% of respondents believed that their training prepared them ‘very well’ or ‘extremely well’ to provide multilingual aphasia services, and only 14.9% associated their knowledge of multilingualism theory with the training they received. It is conceivable that our respondents highlight a clear global gap in SLP training when it comes to concepts related to multilingualism and multilingual aphasia practices. Fig 3B presents SLPs’ responses to Q2.7, which asked which items/themes training programs should emphasize for them to be better prepared to provide clinical services to MPWA, and how important they think these items/themes are. For all listed contents, most respondents rated them as either ‘very important’ or ‘important.’ Respondents considered the content option “treatment for speech and language disorders in MPWA” as the most important topic to be emphasized in professional training, with 51.8% rating it as very important and 21.1% as important. This was followed by the content option “language assessment tools and procedures for MPWA” with 45.7% rating it as very important and 26.3% as important. The content option “Identifying the clinical profile in MPWA” was rated as very important by 40.5% and as important by 28.3%, and the content option “Assessment of premorbid language proficiency in MPWA” was ranked as very important by 33.4% and as important by 32.2%. We also received 17 individual responses under the “other option”, where respondents specified elements, including topics such as codeswitching/mixing, cultural differences, involvement of family or caregivers, and partner training. The full set of results for Q2.5-Q2.7 is available in Appendix 4.

**Fig 4:**
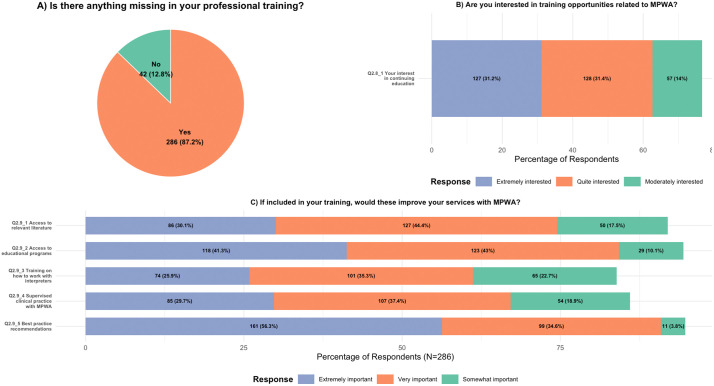
Q2.8 & Q2.9: *SLPs’ responses regarding A) If they believe anything is missing in their training, B) whether they are interested in participating in continuing education, and C) whether they believe that any of these listed elements are included in their training would improve their services to MPWA.* Percentages are rounded. The “other” option and answer options ‘not important’ and ‘not important at all’ are not shown due to very low response rates.

Fig 4 displays the summary of responses to Q2.8 and Q2.9, which asked the respondents whether they were interested in participating in continuing education opportunities and whether they believed any aspects were lacking in their training that would have better prepared them to deliver clinical services to MPWA. The responses to Q2.8 are largely affirmative, with 127 respondents (31.2%) reporting that they are *extremely interested* and 128 respondents (31.4%) indicating that they are *quite interested*. Those who indicated mild or moderate interest or were not interested were in the sheer minority, with a total of 68 respondents (17.7% out of 407 eligible respondents). Q2.9 asked if the SLPs were missing anything in their training that would have prepared them to provide clinical services to MPWA. If they responded ‘yes’, they were asked to respond to an optional question asking them what should have been included in their training (see Figs 4A and 4C). Most respondents (n = 286, 87.2%) selected ’yes’, indicating that they lacked specific training related to managing MPWA. Of those who answered ‘yes’, ‘best practice recommendations to work with MPWA’ was considered to be extremely important (n = 161, 56.3%) and very important (n = 99, 34.6%). This was followed by the option ‘access to educational programs (courses, workshops, etc.),’ which was rated by many to be extremely important (n = 118, 41.3%) or very important (n = 123, 43%). All three other listed options, that is, ‘access to relevant literature’, ‘training on working with interpreters’, and ‘supervised clinical practice’ were also considered to be important (see Fig 4C). Q2.9 also generated a moderate number of “other” responses, with input provided by the participants (n = 15). The input was diverse, mentioning needs such as training clinicians to have working proficiency in multiple languages or dialects, preparing interpreters for clinical contexts, increasing training on cultural differences, and improving access to assessment and treatment materials in languages beyond the national or standard language.

### 3.3. Clinical settings and caseloads

**Fig 5:**
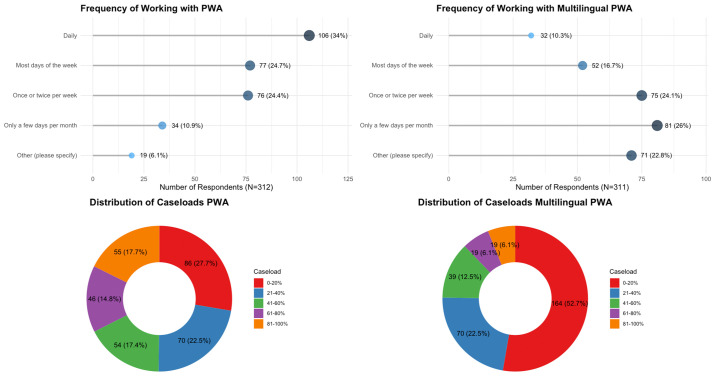
Q3.1-Q3.4: The respondents’ frequency and distribution of caseloads of people with aphasia (PWA, left) and multilingual people with aphasia (MPWA, right).

In this section, we report on the responses to MAP survey questions Q3.1 to Q3.4, which asked about the SLPs’ routine practice with PWA and MPWA, on their caseloads in the clinic. Fig 5 displays this data. We received a total of 312 responses to Q3.1 regarding how frequently SLPs worked with PWA. Of these, 106 (33.9%) reported working with PWA daily, while 77 (24.6%) and 76 (24.3%) indicated working with PWA most days or once/twice per week, followed by 34 (10.9%), who mentioned that they provided care to PWA only a few days per month. Other responses (n = 15) included 9 participants (60%) who reported working with PWA only a few days per year, 5 participants (33.3%) who reported no current clinical practice, and 1 participant who reported no PWA caseload (6.7%). Regarding the distribution of PWA within respondents’ caseloads, 86 (27.7%) reported that PWA makes up 0–20% of their general caseload. This was followed by 21–40% (n = 70, 22.5%), 41–60% (n = 54, 17.4%), 61–80% (n = 46, 14.8%), and 81–100% (n = 55, 17.7%) of their caseload.

We received 311 responses to the questions regarding frequency of MPWA in the clinical workplace, 10.3% (N = 32) reported working with MPWA daily, while 16.7% (N = 52) indicated working with them almost daily. Additionally, 24.1% (N = 75) reported working with MPWA most days of the week, and 26.1% (N = 81) reported working with them once or twice per week. A further 22.8% (N = 71) indicated that they worked with MPWA only a few days per month.

When responding to the question about the percentage of MPWA seen in the clinic per year, of the 311 respondents, a total of 52.7% (n = 164) reported seeing no or few MPWA in their clinic per year (0–20%). The remaining 22.5% (n = 70) indicated that MPWA comprised 21–40% of their aphasia caseload, 12.5% (n = 39) reported 41–60%, and 12.2% (n = 38) reported 61–80% and 81–100%. To this question, 69 participants selected the open text response “other”. Around 60% of these respondents (n = 40/69) reported that they rarely worked with MPWA on a monthly or even yearly basis. Around 18% of the respondents (n = 12/69) reported working with MPWA irregularly according to their caseload. Moreover, at around the time of completing the questionnaire, around 12% of the respondents (n = 8/69) were not currently working with any MPWA. There appear to be variations in the MPWA caseloads across countries. For example, in Singapore, SLPs see MPWAs on a regular basis. Out of the 23 SLP respondents working in Singapore, 72.7% indicated that they saw MPWAs either daily or on most days of the week. However, in Turkey, out of 31 respondents who answered this question, 25.8% reported seeing MPWA either on a daily basis or a few times per week. This figure rose to 57.1% in Switzerland.

## 4. Discussion

This global survey builds on previous country- and region-specific studies, expanding our knowledge on multilingual aphasia practices. It presents the perspectives of 407 SLPs representing over 60 countries across all continents (except Antarctica). Drawing on earlier findings, we anticipated that many SLPs provide clinical services to MPWA despite limited specialized training. Our results are discussed in relation to previous findings and address 1) whether multilingualism is common among SLPs, and whether multiple languages are used in clinical settings when delivering services to MPWA; 2) Whether SLPs have conceptual knowledge and hands-on clinical training for working with MPWA; and 3) Whether SLPs’ clinical settings and caseloads typically include MPWA, and the characteristics of these caseloads.

### 4.1. Demographics of SLP respondents

An international sample of SLPs working with MPWA across many different geographical and linguistic locations and regions of the world participated in this survey study. The respondents were heterogeneous in terms of levels of linguistic and educational background, years of experience, clinical setting, and professional roles.

In line with previous surveys [15–19,21–23], our respondents were predominantly female. Most of them worked full-time as SLPs, and a majority had less than 15 years of experience. Similar to the Norwegian survey, the respondents in this global survey were relatively young, 86% being younger than 50 years old, and most of them had less than 15 years of experience. Contrary to the surveys conducted in the U.S., Canada, and Norway [16–18] but more in line with the results from India [20,21], the education level in this survey was slightly lower, with around 57% holding a master’s degree, compared to 98.4% in the U.S. and 70% in Norway. This probably reflects that the SLP profession requires different educational levels across countries. Compared with all previous surveys, which were conducted in single-country contexts, this survey provides a more diverse demographic profile. It thus extends previous findings rather than replicating them. Moreover, it offers an opportunity to examine multilingual aphasia practices from a global perspective.

### 4.2. Multilingual background of SLP respondents

Our respondents’ linguistic backgrounds varied widely. For instance, in comparison to Centeno [16], who reported only 20% of SLPs in the US to be multilingual, almost 80% of the respondents in this global survey reported being multilingual, most speaking English as both first or second/additional language. However, the critical difference here might lie in the fact that Centeno [16] did not necessarily target SLPs who exclusively work with MPWA. Furthermore, respondents reported using a wide range of languages in clinical practice, often beyond their L1, which aligns with earlier surveys in North America, Europe, and Asia [15–18,21–23]. However, SLPs rated their proficiency relatively low in languages other than their first language, suggesting that multilingual SLPs may have limited ability to provide optimal clinical services in their clients’ languages. It is conceivable that the SLPs may speak the same language as their MPWA, but without optimally sufficient proficiency. This limitation was also reported to be one of the main challenges for SLPs in India, Oceania and the Pacific (e.g., [20,25]). Such a discrepancy between SLPs’ language proficiency and multilingual clients’ needs reflects the reliance on interpreters, MPWAs’ relatives, or SLPs’ multilingual colleagues. Importantly, this discrepancy can produce inequities in clinical care, limited access to linguistically appropriate services, and diagnostic inaccuracies. It may be impractical to expect SLPs to be able to independently deliver evidence-based care in languages that they are not highly proficient in. However, it is both feasible and essential to strengthen their capacity to work effectively with their multilingual clients through professional development opportunities throughout their academic preparation and clinical training, improving training in working with interpreters and the use of telerehabilitation to increase access to optimal care (see [37]). This array of opportunities can include foundational coursework on bilingual language development and cross-linguistic variation, supervised practicum involving multilingual patients, explicit training in the use of interpreters, and the potential of exploiting AI-based translators. Embedding MAP professional development opportunities within pre-service education not only equips future SLPs with the practical tools needed to deliver evidence-based clinical services, but also fosters critical thinking, cultural responsiveness, and collaborative competencies required to navigate multilingual clinical care in an ethical manner.

### 4.3. Conceptual knowledge and training

Despite the global prevalence of multilingual MPWA, our findings reveal a persistent gap in formal education and clinical preparation for SLPs working with MPWA, reflecting all previous surveys [15–18,21–23]. In spite of the longstanding trends that most of the world’s population is multilingual and that the number of multilingual individuals is expected to keep growing, more than half of the respondents in this survey reported that they had received minimal education and training in multilingualism and multilingual aphasia during their studies, often limited to a few hours of lectures. This suggests that multilingualism has been only a minor part of the SLP curricula. However, even these unsatisfactory results indicate that education has a slightly greater focus on multilingualism than were the results of several previous surveys. In Norway, as many as 80% never or rarely attended lectures on multilingualism, and two-fifths of the SLPs had not attended any lectures on multilingual aphasia, which resembles the U.S. results. In India, almost all SLPs (93%) reported a lack of training in working with multilingual clients. In contrast, this global survey showed that around one quarter of the SLPs had completed a dedicated course or more on multilingualism in general, and 10% had completed a full course or more on multilingual aphasia, most of which focused on assessment and rehabilitation of MPWA. Expanding the results of the previous surveys, the MAP survey also shows that 1/3 of the SLPs attempt to fill this education gap themselves, by reading independently about multilingualism and multilingual aphasia, after they graduate. In line with previous findings [15–18,21–23], our respondents also expressed a lack of preparation during their education, with only a minority of the respondents feeling prepared for providing services to MPWA. Close to 90% of the SLPs stated that they felt they missed “something” in their education, and the most chosen topic was best-practice guidelines, followed by access to courses and workshops, access to literature, training on working with interpreters, and supervised clinical practice. In fact, as much as more than 60% of the respondents are very interested in further education to better prepare them to provide clinical services. Based on the previous surveys, this was an expected finding. We therefore invited the respondents to indicate the importance of different aspects of how education could be improved in order to prepare the students better for the reality of a growing multilingual client base. The most important topics for the respondents were intervention and assessment of the languages and linguistic background of MPWA. This inadequate education may be a major barrier to equitable service delivery. Despite a growing body of studies on the importance of a comprehensive assessment of all languages and documenting the language history of multilingual individuals (e.g., [38,39,40]), there are no consensual best-practice guidelines to deliver clinical services to MPWA, either for how best to assess language difficulties, or for clinical choices regarding language interventions. Our forthcoming paper [31] outlines SLPs’ current practices regarding these topics in detail. The fact that SLPs need to continue their education independently after obtaining the SLP degree, and also the desire for continuing education opportunities reported by many respondents, indicate shortcomings in the education. There seems to be a critical gap between clinical demands and educational preparation, with implications for both academic programs and continuing education initiatives.

### 4.4. Clinical settings and caseload

The survey revealed considerable variability in how often SLPs work with aphasia in general, with only 1/3 working with aphasia daily. There was even greater variability in the workload of MPWA within SLPs’ caseloads. While some respondents reported daily contact with MPWA, others encountered multilingual clients only occasionally. Frequency in delivering clinical services to MPWA varied across SLPs’ overall caseloads from every day to almost none. This may have been caused by a number of reasons. In countries/regions where SLPs do not see many MPWA as in their region multilingualism may not be predominant, or SLPs do not feel competent enough to provide clinical services to MPWA for different reasons including language barriers and hence they do not accept them in their caseload. On the contrary, in certain regions/countries, such as Singapore or Malaysia, multilingualism is the default, and as a result, virtually all caseload is filled with MPWA. Inevitably, SLPs may not have sufficient time to update their knowledge or develop specialized skills in MPWA. This seems to be the case for several of the previous surveys, and even in multilingual countries or regions, such as India or Canada, the proportion of multilingual clients may also be relatively low [17,20,21]. In Singapore, whose SLPs see MPWAs on a regular basis, with about 70% indicating that they saw MPWA, either daily or on most days of the week, and that 56.5% reported that MPWA make up at least 40% of their aphasia caseloads. This variability in the clinical caseloads across countries has significant implications. Clinicians who rarely work with MPWA must be prepared to manage the complexity of their multilingual clients, even if these are encountered rarely. Therefore, there is a need for flexible, accessible resources and supervised clinical practice to equip SLPs for the work with this client group.

### 4.5. Limitations

There are limitations to this study that should be acknowledged. Despite our efforts to distribute the survey through the CATs network, social media, and SLP organizations across the globe, and although we managed to collect responses from over 60 countries, we did not manage to obtain representative numbers across nations and regions. Some regions are highly underrepresented, especially the African and South American countries, which limits the generalizability of the findings. This is somehow a reflection of the unhidden reality, as linguistic diversity is very high in those areas – nonetheless, the availability of trained SLPs is very poor. While we translated the survey into 14 languages, the survey was not available in all languages in which the SLPs reported to providing clinical services, which possibly prevented the recruitment of SLPs in some regions. SLP organizations are not available in all countries, and even in countries where an organization is in place, many SLPs are not necessarily part of it, creating a challenge to reach out to target survey participants. We should also mention that SLP training is vastly variable, while in some countries training is only available at the postgraduate level, in some others, certificate or associate degree programs are offered. This has likely caused a level of heterogeneity in our data. Also importantly, the convenience sampling may have disproportionately attracted those SLPs who are already interested in multilingual issues surrounding aphasia rehabilitation, providing us with a limited view of the larger context of clinical practice involving MPWA. A further possible limitation of the study is that there may be a self-report bias, as some participants may have overestimated or underestimated their multilingual proficiency and competence in delivering clinical care in multilingual contexts. Self-report measures may inevitably be affected by biases such as social desirability or underestimation of one’s own abilities, which could influence the accuracy of the reported levels of proficiency and experience. Consequently, the findings should be interpreted with caution, as the reported competencies may not fully reflect objectively measured skills or clinical performance.

### 4.6. Implications for clinical practice and future directions

Our international survey results confirm the findings of previous country-specific surveys that most SLPs do not feel properly prepared for their clinical work with MPWA. Thus, this is a global trend that has not improved over the last decade. Our findings highlight an overdue need for SLP education to strengthen multilingual competence in clinical aphasia care. SLP programs should include clinical work with multilingual individuals in general and MPWA in particular, as part of their education, to equip SLPs to better provide culturally responsive care. To address these gaps, international collaborations need to establish global best-practice guidelines for multilingual aphasia care and to ensure the availability of continuing education. Future research should also explore innovative approaches, such as telepractice, to overcome resource limitations. As there is a global trend of children and young adults speaking multiple languages, the likelihood of encountering MPWA in the future should increase. This creates a genuine concern if, worldwide, SLPs continue to lack appropriate training in assessing and treating MPWAs. Our international survey results based on 407 SLPs, therefore constitute a timely call for concrete action.

## Supporting information

S1 Appendix Full list of respondents’ countries of practice and training. There was a total of 402 respondents who indicated their country of training and 406 indicated their country of clinical practice. A value of 0% indicates that no responses were received from that country.
(DOCX)

S2 Appendix The top 10 languages spoken by respondents while delivering clinical services to MPWA are ranked by frequency of use over the past five years, ranging from the most frequently used (Language 1) to the least frequently used language/dialect (Language 4/Other). For “Others”, participants also provided a largely variable responses including, “rudimentary” Portuguese, Italian, Albanian, Polish, Hungarian, and Finnish, or the respondents’ willingness to learn MPWA’s language in whichever that they can provide their services with including Swedish, Russian, Ukrainian, Lithuanian, German. Few participants provided additional information regarding dialects (e.g., Arabic – Levantine; Greek – Cypriot).
(DOCX)

S3 Appendix Percentage of responses to Q2.5, content of courses that the SLPs had received on multilingualism or multilingual aphasia, across all code categories reflecting the content of courses attended by survey respondents. (DOCX)

S4 Appendix Full data tables summarizing frequency and percent responses in Likert-scale questions Q2.4 through Q2.7 are given below. Table A4_1. Section Q2.4 and Q2.5.
(DOCX)

S1 File MAP Survey Final Nov 2022. (PDF)

## References

[1] Ardila A, Ramos E. Bilingualism in the contemporary world. Speech and language disorders in bilinguals. 2007.

[2] Grosjean F. Bilingual: Life and reality. Harvard University Press; 2010.

[3] IALP. International Association of Logopedics and Phoniatrics. 2025. Available from: https://ialpglobal.org/aphasia/

[4] Centeno JG, Kiran S, Armstrong E. Aphasia management in growing multiethnic populations. Taylor & Francis; 2020.

[5] Centeno JG. Serving bilingual patients with aphasia: challenges, foundations, and procedures. Rev Logop Foniatr Audiol. 2009;29(1):30–6. doi: 10.1016/s0214-4603(09)70141-x

[6] Mellahn K, Larkman C, Lakhani A, Siyambalapitiya S, Rose ML. The nature of inpatient rehabilitation for people with aphasia from culturally and linguistically diverse backgrounds: a scoping review. Top Stroke Rehabil. 2023;30(2):146–56. doi: 10.1080/10749357.2021.2008599 34854368

[7] Larkman CS, Mellahn K, Han W, Rose ML. Aphasia rehabilitation when speech pathologists and clients do not share the same language: a scoping review. Aphasiology. 2022;37(4):635–57. doi: 10.1080/02687038.2022.2035672

[8] Arslan S, Peñaloza C. Across countries and cultures: the assessment of aphasia in linguistically diverse clinical populations. Taylor & Francis; 2025.

[9] Larkman CS, Lanyon L, Rose ML. “It’s so complicated”: a qualitative study of interpreters’ experiences working with speech pathologists to support the provision of aphasia rehabilitation. Disabil Rehabil. 2025;47(14):3637–48. doi: 10.1080/09638288.2024.2435518 39628319

[10] Goral M, Conner PS. Language Disorders in Multilingual and Multicultural Populations. Annu Rev Appl Linguist. 2013;33:128–61. doi: 10.1017/S026719051300010X 26257455 PMC4527602

[11] Ansaldo AI, Saidi LG. Aphasia therapy in the age of globalization: cross-linguistic therapy effects in bilingual aphasia. Behav Neurol. 2014;2014:603085. doi: 10.1155/2014/603085 24825963 PMC4006602

[12] Goral M, Hejazi Z. Aphasia in Multilingual Patients. Curr Neurol Neurosci Rep. 2021;21(11):60. doi: 10.1007/s11910-021-01148-5 34674041

[13] Scimeca M, Abdollahi F, Peñaloza C, Kiran S. Clinical perspectives and strategies for confronting disparities in social determinants of health for Hispanic bilinguals with aphasia. J Commun Disord. 2022;98:106231. doi: 10.1016/j.jcomdis.2022.106231 35688011 PMC9228944

[14] Matić A, Pourquié M, Norvik M, Kuvač Kraljević J, Gram Simonsen H, Martínez-Ferreiro S, et al. Setting a research agenda for the assessment and treatment of aphasia in minority languages. Cortex. 2026;198:13–26. doi: 10.1016/j.cortex.2026.02.013 41791212

[15] Centeno JG. Issues and principles in service delivery to communicatively impaired minority bilingual adults in neurorehabilitation. Seminars in Speech and Language. Thieme Medical Publishers; 2009. 10.1055/s-0029-1225951 19711232

[16] Centeno JG. Assessing services with communicatively impaired bilingual adults in culturally and linguistically diverse neurorehabilitation programs. J Commun Disord. 2015;58:58–73. doi: 10.1016/j.jcomdis.2015.10.005 26513217

[17] D’Souza C. Survey of Canadian speech-language pathology service delivery to linguistically diverse clients. Dalhousie University; 2010.

[18] Norvik MI, Lind M, Jensen BU. Working with multilingual aphasia: attitudes and practices among speech and language pathologists in Norway. Int Multiling Res J. 2022;16(4):273–90. doi: 10.1080/19313152.2021.2015935

[19] Pourquié M. Enquête sur les pratiques orthophoniques vis à vis du plurilinguisme. Lapurdum. 2023;24:279–94. doi: 10.4000/127u6

[20] Niharika MK, Gurkar Harshitha HN, Aishwarya SY, Suman S. Speech-language pathologists’ perspectives on bilingual service delivery in India: a preliminary survey. Speech Lang Hear. 2023;27(2):79–86. doi: 10.1080/2050571x.2023.2259145

[21] Chandana SSM, Goswami SP. Survey on current practices in the clinical assessment of persons with aphasia in India. Speech Lang Hear. 2025;28(1):2539572.

[22] Guo YE, Togher L, Power E. Speech pathology services for people with aphasia: what is the current practice in Singapore?. Disabil Rehabil. 2014;36(8):691–704. doi: 10.3109/09638288.2013.804597 23786347

[23] Hassan FH, Lee GZH, A Razak R, A Aziz MA, Joginder Singh S. The management of multilingual adults with aphasia in Malaysia: current practices, needs, and challenges. Aphasiology. 2023;38(3):487–509. doi: 10.1080/02687038.2023.2214299

[24] Sara Jodache SJ, Tami Howe TH, Samantha Siyambalapitiya SS. “Are we…providing them with an equal service?”: Speech-language pathologists’ perceptions of bilingual aphasia assessment of Samoan-English speakers. Clin Arch Commun Disord. 2019;4(1):41–51. doi: 10.21849/cacd.2019.00024

[25] Siyambalapitiya S, Davidson B. Managing aphasia in bilingual and culturally and linguistically diverse individuals in an Australian context. J Clin Pract Speech-Lang Pathol. 2015;17(1):13–9.

[26] Roger P, Code C, Sheard C. Assessment and management of aphasia in a linguistically diverse society. Asia Pac J Speech Lang Hear. 2000;5(1):21–34. doi: 10.1179/136132800807547573

[27] Quique YM, Kong AP-H, Owusu Antwi AA, Jagoe C. Language rights and publication practices in aphasia research: lessons learned from developing aphasia assessments in multiple languages. Aphasiology. 2025;:1–18. doi: 10.1080/02687038.2025.2508444

[28] Penn C. Asking new questions and seeking new answers: The reality of aphasia practice in South Africa. Top Lang Disord. 2014;34(2):168–81.

[29] Beveridge MEL, Bak TH. The languages of aphasia research: Bias and diversity. Aphasiology. 2011;25(12):1451–68. doi: 10.1080/02687038.2011.624165

[30] Egia-Zabala M, Munarriz-Ibarrola A. Language Diversity and Bi/Multilingualism in Aphasia Research. Languages. 2024;9(10):325.

[31] Norvik MI, Arslan S, Alyahya RSW, Anjum J, Fyndanis V, Grima R, et al. Common practices and current challenges for speech-language pathologists working with multilingual people with aphasia: an international survey. forthcoming.

[32] Eysenbach G. Improving the quality of web surveys: the checklist for reporting results of internet e-surveys (cherries). J Med Internet Res. 2004;e34. 10.2196/jmir.6.3.e34 PMC1550605 15471760

[33] Willis GB, Lessler JT. Question appraisal system QAS-99. National Cancer Institute; 1999.

[34] Cochran WG. Sampling techniques. Johan Wiley & Sons Inc.; 1977.

[35] Wickham H. dplyr: A grammar of data manipulation. R package version 04. 2015. 156 p.

[36] Wickham H, Vaughan D, Girlich M. tidyr: Tidy Messy Data. R package version 1.1. 3. CRAN R-project org/package= tidyr. 2020.

[37] Peñaloza C, Scimeca M, Gaona A, Carpenter E, Mukadam N, Gray T, et al. Telerehabilitation for Word Retrieval Deficits in Bilinguals With Aphasia: Effectiveness and Reliability as Compared to In-person Language Therapy. Front Neurol. 2021;12:589330. doi: 10.3389/fneur.2021.589330 34093382 PMC8172788

[38] Sung JE, Scimeca M, Li R, Kiran S. Cross-Linguistic and Multicultural Considerations in Evaluating Bilingual Adults With Aphasia. Am J Speech Lang Pathol. 2024;33(6):2716–31. doi: 10.1044/2024_AJSLP-23-00496 39196846 PMC11546902

[39] Kuzmina E, Goral M, Norvik M, Weekes BS. What Influences Language Impairment in Bilingual Aphasia? A Meta-Analytic Review. Front Psychol. 2019;10:445. doi: 10.3389/fpsyg.2019.00445 31024369 PMC6460996

[40] Arslan S, Felser C. Comprehension of wh-questions in Turkish-German bilinguals with aphasia: A dual-case study. Clin Linguist Phon. 2018;32(7):640–60. doi: 10.1080/02699206.2017.1416493 29271669

