# Supplementary material for: Characterizing the linguistic profiles, training needs, and caseloads of speech language pathologists providing clinical services to multilingual people with aphasia: The international Multilingual Aphasia Practices (MAP) consensus group survey
Source: PLoS One. 2026 Apr 9;21(4):e0346488. doi: 10.1371/journal.pone.0346488 (PMC13065022; doi:10.1371/journal.pone.0346488)
Supplement: S1 Appendix — There was a total of 402 respondents who indicated their country of training and 406 indicated their country of clinical practice. A value of 0% indicates that no responses were received from that country. (DOCX) [file pone.0346488.s001.docx]

**Appendix 1. Full list of respondents’ countries of practice and training. There was a total of 402 respondents who indicated their country of training and 406 indicated their country of clinical practice. A value of 0% indicates that no responses were received from that country.**

| **Country** | **Country of training** | **Country of practice** | **Country** | **Country of training** | **Country of practice** | **Country** | **Country of training** | **Country of practice** |
| --- | --- | --- | --- | --- | --- | --- | --- | --- |
| Switzerland | 30 (7.46%) | 43 (10.31%) | USA | 29 (7.21%) | 24 (5.76%) | Turkey | 28 (6.97%) | 32 (7.67%) |
| Australia | 27 (6.72%) | 14 (3.36%) | UK | 26 (6.47%) | 16 (3.84%) | Spain | 25 (6.22%) | 26 (6.24%) |
| Sweden | 19 (4.73%) | 19 (4.56%) | Norway | 17 (4.23%) | 17 (4.08%) | India | 15 (3.73%) | 14 (3.36%) |
| France | 12 (2.99%) | 14 (3.36%) | Germany | 12 (2.99%) | 12 (2.88%) | Ireland | 11 (2.74%) | 10 (2.40%) |
| Malta | 11 (2.74%) | 11 (2.64%) | Canada | 10 (2.49%) | 9 (2.16%) | Cyprus | 10 (2.49%) | 13 (3.12%) |
| Morocco | 9 (2.24%) | 10 (2.40%) | Singapore | 9 (2.24%) | 26 (6.24%) | Belgium | 8 (1.99%) | 5 (1.20%) |
| Greece | 8 (1.99%) | 9 (2.16%) | Italy | 8 (1.99%) | 4 (0.96%) | Saudi Arabia | 7 (1.74%) | 7 (1.68%) |
| Croatia | 6 (1.49%) | 6 (1.44%) | Jordan | 6 (1.49%) | 4 (0.96%) | Netherlands | 5 (1.24%) | 3 (0.72%) |
| South Africa | 5 (1.24%) | 5 (1.20%) | Denmark | 4 (1.00%) | 4 (0.96%) | Ghana | 4 (1.00%) | 6 (1.44%) |
| Portugal | 3 (0.75%) | 2 (0.48%) | Afghanistan | 2 (0.50%) | 0 (0%) | Antigua Barbuda | 2 (0.50%) | 2 (0.48%) |
| Armenia | 2 (0.50%) | 2 (0.48%) | Austria | 2 (0.50%) | 1 (0.24%) | Finland | 2 (0.50%) | 2 (0.48%) |
| Israel | 2 (0.50%) | 2 (0.48%) | Lebanon | 2 (0.50%) | 4 (0.96%) | Philippines | 2 (0.50%) | 2 (0.48%) |
| Slovenia | 2 (0.50%) | 3 (0.72%) | Uruguay | 2 (0.50%) | 3 (0.72%) | China | 2 (0.50%) | 2 (0.48%) |
| Albania | 1 (0.25%) | 2 (0.48%) | Argentina | 1 (0.25%) | 0 (0%) | Bulgaria | 1 (0.25%) | 2 (0.48%) |
| Chile | 1 (0.25%) | 0 (0%) | Colombia | 1 (0.25%) | 0 (0%) | Costa Rica | 1 (0.25%) | 1 (0.24%) |
| Egypt | 1 (0.25%) | 1 (0.24%) | Hong Kong | 1 (0.25%) | 1 (0.24%) | Iceland | 1 (0.25%) | 3 (0.72%) |
| Latvia | 1 (0.25%) | 1 (0.24%) | Lithuania | 1 (0.25%) | 1 (0.24%) | Malaysia | 1 (0.25%) | 1 (0.24%) |
| Pakistan | 1 (0.25%) | 1 (0.24%) | Russia | 1 (0.25%) | 1 (0.24%) | South Korea | 1 (0.25%) | 0 (0%) |
| Suriname | 1 (0.25%) | 1 (0.24%) | Kenya | 0 (0%) | 4 (0.96%) | UAE | 0 (0%) | 2 (0.48%) |
| Niger | 0 (0%) | 1 (0.24%) | Zimbabwe | 0 (0%) | 1 (0.24%) | Ethiopia | 0 (0%) | 1 (0.24%) |
| Georgia | 0 (0%) | 1 (0.24%) | Oman | 0 (0%) | 1 (0.24%) | Tanzania | 0 (0%) | 1 (0.24%) |
| Bahrain | 0 (0%) | 1 (0.24%) | — | — | — | — | — | — |
