# Supplementary material for: Characterizing the linguistic profiles, training needs, and caseloads of speech language pathologists providing clinical services to multilingual people with aphasia: The international Multilingual Aphasia Practices (MAP) consensus group survey
Source: PLoS One. 2026 Apr 9;21(4):e0346488. doi: 10.1371/journal.pone.0346488 (PMC13065022; doi:10.1371/journal.pone.0346488)
Supplement: S2 Appendix — For “Others”, participants also provided a largely variable responses including, “rudimentary” Portuguese, Italian, Albanian, Polish, Hungarian, and Finnish, or the respondents’ willingness to learn MPWA’s language in whichever that they can provide their services with including Swedish, Russian, Ukrainian, Lithuanian, German. Few participants provided additional information regarding dialects (e.g., Arabic – Levantine; Greek – Cypriot). (DOCX) [file pone.0346488.s002.docx]

**Appendix 2. The top 10 languages spoken by respondents while delivering clinical services to MPWA are ranked by frequency of use over the past five years, ranging from the most frequently used (Language 1) to the least frequently used language/dialect (Language 4/Other). For “Others”, participants also provided a largely variable responses including, “rudimentary” Portuguese, Italian, Albanian, Polish, Hungarian, and Finnish, or the respondents’ willingness to learn MPWA’s language in whichever that they can provide their services with including Swedish, Russian, Ukrainian, Lithuanian, German. Few participants provided additional information regarding dialects (e.g., Arabic - Levantine; Greek - Cypriot).**

| ***Sl. No.*** | **Language 1 (% Makeup)** | **Language 2 (% Makeup)** | **Language 3 (% Makeup)** | **Language 4 (% Makeup)** | **Others**  **(% Makeup)** |
| --- | --- | --- | --- | --- | --- |
| ***1*** | English (27.46%) | English (45.14%) | English (21.9%) | English (22.92%) | English (6.06%) |
| ***2*** | French (9.07%) | Spanish (8.69%) | Italian (13.87%) | French (12.5%) | German (6.06%) |
| ***3*** | Turkish (8.06%) | French (6.94%) | French (8.03%) | Spanish (10.42%) | Italian (6.06%) |
| ***4*** | Spanish (5.54%) | Mandarin* (6.6%) | Spanish (8.03%) | German (6.25%) | Polish (6.06%) |
| ***5*** | Swedish (5.03%) | German (5.21%) | German (5.84%) | Italian (6.25%) | Russian (6.06%) |
| ***6*** | Arabic (4.78%) | Basque (3.47%) | Cantonese* (5.1%) | Hokkien* (4.17%) | Spanish (6.06%) |
| ***7*** | Norwegian (4.53%) | Russian (2.08%) | Hokkien* (2.92%) | Malay (4.17%) | Albanian (3.03%) |
| ***8*** | Greek (4.28%) | Afrikaans (1.74%) | Mandarin* (2.92%) | Hakka* (2.08%) | Bosnian(3.03%) |
| ***9*** | German (3.79%) | Kiswahili (1.74 %) | Ga (2.19%) | High German (2.08%) | Cantonese* (3.03%) |
| ***10*** | Italian (3.02%) | Arabic (1.39%) | Malay (2.19%) | Hindi (2.08%) | Finnish (3.03%) |
| ***Total*** | A total of 300 (75.57%) responses represented out of 397 (100%) responses. | A total of 239 (82.99%) responses represented out of 288 (100%) responses. | A total of 100 (72.99%) responses represented out of 137 (100%) responses. | A total of 35 (72.92%) responses represented out of 48 (100%) responses. | A total of 16 (48.48%) responses represented out of 33 (100%) responses. |
| *A dialect of the Chinese language. | | | | | |
