# Supplementary material for: Characterizing the linguistic profiles, training needs, and caseloads of speech language pathologists providing clinical services to multilingual people with aphasia: The international Multilingual Aphasia Practices (MAP) consensus group survey
Source: PLoS One. 2026 Apr 9;21(4):e0346488. doi: 10.1371/journal.pone.0346488 (PMC13065022; doi:10.1371/journal.pone.0346488)
Supplement: S3 Appendix — (DOCX) [file pone.0346488.s003.docx]

**Appendix 3.** **Percentage of responses to Q2.5, content of courses that the SLPs had received on multilingualism or multilingual aphasia, across all code categories reflecting the content of courses attended by survey respondents.**

##
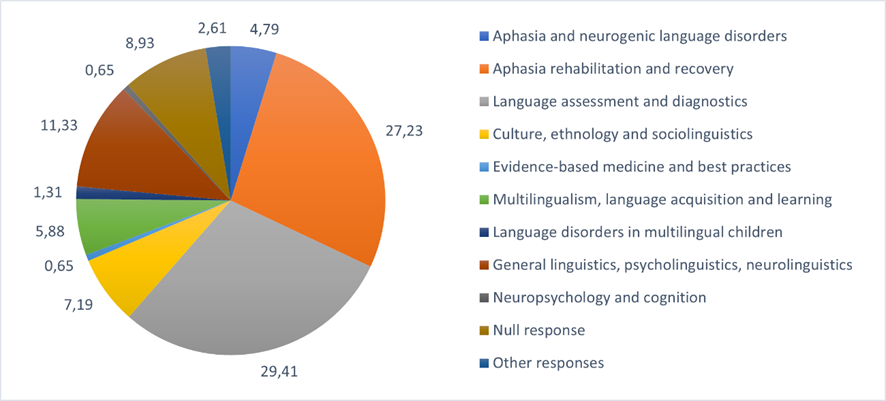


## When splitting down the multiple word responses, the total of unique responses raised to 459 (8.93% were null responses). Most participant responses reflected training on *language assessment and diagnostics* (29.41%) followed by *aphasia rehabilitation and recovery* (27.23%) and *general linguistics, psycholinguistics and neurolinguistics* (11.33%). Less frequent responses were distributed across the following categories: *culture, ethnology and sociolinguistics* (7.19%); *multilingualism, language acquisition and learning* (5.88%); *aphasia and neurogenic language disorders* (4.79%); *language disorders in multilingual children* (1.31%); *evidence-based medicine and best practices* (0.65%) and, *neuropsychology* *and cognition* (0.65%). Stand-alone responses which could not be classified into the established codes were grouped into a category for *other responses* (2.61%).
