# Supplementary material for: Characterizing the linguistic profiles, training needs, and caseloads of speech language pathologists providing clinical services to multilingual people with aphasia: The international Multilingual Aphasia Practices (MAP) consensus group survey
Source: PLoS One. 2026 Apr 9;21(4):e0346488. doi: 10.1371/journal.pone.0346488 (PMC13065022; doi:10.1371/journal.pone.0346488)
Supplement: S4 Appendix — Table A4_1. Section Q2.4 and Q2.5. (DOCX) [file pone.0346488.s004.docx]

**Appendix 4. Full data table summarizing frequency and percent responses in Likert-scale questions Q2.4 through Q2.7 are given below. Table A4_1. Section Q2.4 and Q2.5**

|  |  | *Q2.4 Which of the following options best describes any formal academic or clinical training you have received on bi/multilingualism (not specific to aphasia)?* | | *Q2.5 Which of the following options best describes any formal academic or clinical training you have received on bi/multilingual aphasia?* | |
| --- | --- | --- | --- | --- | --- |
| *Option* | *Response* | *N* | *%* | *N* | *%* |
| Few classes | During | 105 | 25.8 | 91 | 22.3 |
|  | After | 26 | 6.3 | 24 | 5.8 |
|  | Both | 16 | 3.9 | 9 | 2.2 |
| Few hours | During | 35 | 8.6 | 36 | 8.8 |
|  | After | 128 | 31.4 | 107 | 26.3 |
|  | Both | 55 | 13.5 | 32 | 7.8 |
| Independent reading | During | 50 | 12.3 | 42 | 13.3 |
|  | After | 137 | 33.6 | 131 | 32.1 |
|  | Both | 97 | 23.8 | 66 | 16.2 |
| More than a full course | During | 35 | 8.6 | 16 | 3.9 |
|  | After | 19 | 4.6 | 20 | 4.9 |
|  | Both | 8 | 1.9 | 7 | 1.7 |
| Not received any | During | 67 | 16.4 | 84 | 20.6 |
|  | After | 49 | 12.1 | 47 | 11.5 |
|  | Both | 38 | 9.3 | 50 | 12.2 |
| One full course | During | 60 | 14.7 | 26 | 6.3 |
|  | After | 26 | 6.3 | 17 | 4.1 |
|  | Both | 5 | 1.2 | 1 | 0.2 |

**Table A4_2. Section Q2.6**

|  | *Q2.6_1 How well has training prepared SLP for assessment?* | | *Q2.6_2 How well has training prepared SLP for therapy?* | | *2.6_3 How well familiarized with theories of bi/multilingualism?* | |
| --- | --- | --- | --- | --- | --- | --- |
| *Response* | *N* | *%* | *N* | *%* | *N* | *%* |
| Extremely well | 17 | 4.177 | 14 | 3.440 | 7 | 1.720 |
| Very well | 45 | 11.057 | 39 | 9.582 | 61 | 14.988 |
| Somewhat well | 108 | 26.536 | 100 | 24.570 | 133 | 32.678 |
| Not so well | 88 | 21.622 | 95 | 23.342 | 86 | 21.130 |
| Not well at all | 57 | 14.005 | 67 | 16.462 | 28 | 6.880 |

**Table A4_3. Sections Q2.7**

| What training programs should emphasize? | *Q2.7_1 Multilingual language acquisition and learning* | | *Q2.7_2Working with interpreters or translators* | | *2.7_3 Assessment of cognition in MPWA?* | | *2.7_4 Assessment of premorbid language proficiency in M PWA* | | *2.7_5 Assessment and treatment of minority/heritage* | | *2.7_6 Language assessment tools and procedures for MPWA* | |
| --- | --- | --- | --- | --- | --- | --- | --- | --- | --- | --- | --- | --- |
| *Response* | *N* | *%* | *N* | *%* | *N* | *%* | *N* | *%* | *N* | *%* | *N* | *%* |
| **Very important** | 121 | 29.7 | 105 | 25.8 | 127 | 31.2 | 136 | 33.4 | 104 | 25.6 | 186 | 45.7 |
| **Important** | 121 | 29.7 | 110 | 27.0 | 124 | 30.5 | 131 | 32.2 | 126 | 31.0 | 107 | 26.3 |
| **Somewhat important** | 56 | 13.8 | 74 | 18.2 | 52 | 12.8 | 34 | 8.4 | 70 | 17.2 | 16 | 3.9 |
| **Somewhat unimportant** | 13 | 3.2 | 20 | 4.9 | 5 | 1.2 | 8 | 2.0 | 11 | 2.7 | 3 | 0.7 |
| **Very unimportant** | 7 | 1.7 | 4 | 1.0 | 5 | 1.2 | 4 | 1.0 | 4 | 1.0 | 5 | 1.2 |

***Table A4_4.*** *Sections Q2.7 continued.*

| What training programs should emphasize? | *Q2.7_7 Diverse patterns of language impairment* | | *Q2.7_8 Identifying the clinical profile in MPWA* | | *2.7_9 Research on MPWA that informs clinical practice* | | *2.7_10 Treatment for speech language disorders in MPWA* | | *2.7_11 Other* | |
| --- | --- | --- | --- | --- | --- | --- | --- | --- | --- | --- |
| *Response* | *N* | *%* | *N* | *%* | *N* | *%* | *N* | *%* | *N* | *%* |
| **Very important** | 140 | 34.4 | 165 | 40.5 | 156 | 38.3 | 211 | 51.8 | 17 | 4.2 |
| **Important** | 135 | 33.2 | 115 | 28.3 | 119 | 29.2 | 86 | 21.1 | 11 | 2.7 |
| **Somewhat important** | 31 | 7.6 | 27 | 6.6 | 30 | 7.4 | 13 | 3.2 | 6 | 1.5 |
| **Somewhat unimportant** | 2 | 0.5 | 4 | 1.0 | 4 | 1.0 | 1 | 0.2 | 2 | 0.5 |
| **Very unimportant** | 5 | 1.2 | 5 | 1.2 | 5 | 1.2 | 5 | 1.2 | 4 | 1.0 |
