# Supplementary material for: Characterizing the linguistic profiles, training needs, and caseloads of speech language pathologists providing clinical services to multilingual people with aphasia: The international Multilingual Aphasia Practices (MAP) consensus group survey
Source: PLoS One. 2026 Apr 9;21(4):e0346488. doi: 10.1371/journal.pone.0346488 (PMC13065022; doi:10.1371/journal.pone.0346488)
Supplement: S1 File — (PDF) [file pone.0346488.s005.pdf]

MAP - Multilingual Aphasia Practices

WORKING WITH BI/MULTILINGUAL PEOPLE WITH APHASIA: COMMON PRACTICES AND CURRENT CHALLENGES FOR SPEECH-LANGUAGE THERAPISTS

Purpose of the study

This survey is conducted by the Multilingual Aphasia Practice Group (Working group 2: *Aphasia Assessment and Outcomes*), which is part of the international network *Collaboration of Aphasia Trialists* (https://www.aphasiatrials.org/). The research work conducted by this group is focused on a variety of issues regarding multilingualism and aphasia.

Culturally and linguistically diverse societies require Speech Language Therapists/Pathologists (SLT/SLPs) to provide clinical services to bi/multilingual speakers. However, SLT/SLPs who work with people with aphasia may not always be specifically or sufficiently trained to work in such bi/multilingual contexts and often lack tools, knowledge and/or experience to provide linguistically and culturally appropriate clinical services (e.g., for the assessment or the treatment of bi/multilingual people with aphasia) in more than one language. This survey aims:

- To assess the degree of awareness of professional Speech-Language Therapists/Pathologists (SLTs/SLPs) about bi/multilingualism in their clinical practice.
- To determine the frequency of their clinical practice with bi/multilingual people with aphasia and their perceptions of readiness for the assessment and treatment of this population.
- To identify their common clinical practices and perceived barriers when providing clinical services to bi/multilingual people with aphasia.

What do we expect from you?

You can only participate in this study if you are a speech and language therapist/pathologist (SLT/SLPs) who provides services to adults with aphasia.

You are invited to complete this online survey with an estimated duration of 15-25 minutes via Qualtrics XM. You may take your time to respond to the questions as there is no time limit. Please respond to all questions and make sure they reflect your views and experiences as much as possible.

In the first and second part of this survey, you will find questions regarding demographic information (i.e., your age, gender, education, employment). Please rest assured that your responses will be kept completely confidential. In the third and fourth sections, we will request your opinion about multilingual aphasia assessment and common practices in treatment relevant to your country/region. These questions intend to compile common multilingual aphasia practices across different countries, not to test your knowledge.

This survey is available in a number of languages including (but not limited to) English, Spanish, Turkish, French, Norwegian, Greek, Basque, Arabic, Mandarin Chinese, and Galician. Please choose the language that you are **more comfortable** with to complete the survey.

Voluntary nature of participation, compensation, and benefit
Your participation in this study is voluntary. You have the right to choose to participate or to refuse participation. You will not receive any financial compensation for your participation. The survey will help identify strengths, limitations and current gaps in the clinical practice of SLPs/SLTs with bi/multilingual people with aphasia and will inform educational and training programs for SLPs/SLTs to improve their professional practice with this population. Given its potential benefits, we would hope to see you participate in this survey.

Confidentiality of the research data
We will not record any data leading to the identification of the survey participants (i.e. names, phone numbers, IP numbers, or other electronic

identifiers). The personal data collected in this study is limited to age, gender, education level, employment status and alike with the only purpose of having a better understanding and interpretation of the survey responses. Personal data will not be traceable. Each participant will be assigned a code number at survey completion so their personal data and responses are processed confidentially and anonymously. All personal data will be stored separately from survey responses.

We will anonymously store and process your data, however, we may use the data for scientific publications and/or dissemination (such as conference talks, workshops for SLPs/SLTs, or lectures to train students). YOUR PERSONAL DATA WILL NEVER BE DISCLOSED. The collected data will be stored electronically at the University of Groningen cloud drive with password protection. Public or third-party access to this data will not be given.

For questions regarding this survey, please contact investigators at: catswg2@univ-cotedazur.fr
For questions regarding research ethics, please contact the Research Ethical Review Committee (CETO) of the Faculties of Arts, Philosophy, and Theology and Religious Studies of the University of Groningen, e-mail: ceto@rug.nl

---

*

Consent 1 Are you a professional SLT/SLP?

- ◯ Yes
- ◯ No

---

*

Consent 2 Do you provide / Have you provided clinical services (assessment, treatment, etc.) to people with aphasia?

○ Yes

○ No

*

By giving my explicit consent below, I acknowledge that:

My participation in this survey is voluntary.
Completing and submitting the survey constitutes consent.
I am 18 years of age or older and a trained SLT/SLP professional.
I have been sufficiently informed about the study.
I understand that my data will be treated anonymously and stored in a protected non-public server.

○ I consent to participate

○ I do not consent to participate

End of Block: Information and Consent

Start of Block: SECTION 1: DEMOGRAPHIC INFORMATION

SECTION 1 SECTION 1: DEMOGRAPHIC INFORMATION

Q1.1 Please select your age group

Q1.2 Please select the gender you identify yourself with

- Male
- Female
- Non-binary
- Not listed

Q1.3 Please indicate your current employment status

- Full-time
- Part-time
- Unemployed
- Retired
- Other: please specify

Q1.4 Please indicate the number of years of working experience you have as a SLT/SLPs

- 1-5
- 6-10
- 11-15
- >16

Q1.5 We consider bi/multilingual speakers as individuals who speak and understand two or more languages. Which one of the following statements describes best your linguistic abilities?

○ I consider myself a bi/multilingual person: I speak and understand more than one language

(Which languages? please list in the order of proficiency)
____

○ I consider myself a monolingual person: I speak and understand only one language.

X→

Q1.6 Please indicate your country of clinical practice (You can select multiple countries as appropriate).

Q1.7 Which language(s) and/or dialect(s) do you use to communicate with **people with aphasia**? Please state each language/dialect in the order of frequency of use in the last five years (Examples, Language 1: Norwegian; Language 2: Quebec French; Language 3: Guarani etc.).

○ Language 1 ____

○ Language 2 ____

○ Language 3 ____

○ Language 4 ____

○ Others (please list) ____

Q1.8 Rate your perceived proficiency to provide clinical services (e.g.: assessment, treatment) in each one of the languages/dialects you use in your clinical practice using the following 5-point scale from VERY LOW (point 1) to VERY HIGH (point 5) type in the languages/dialects in each row (e.g. Language 1: English; Language 2: Spanish; Language 3: Guarani etc.). - Language 1

	1 Very low	2 Low	3 Medium	4 High	5 Very high
Language 1	○	○	○	○	○

Language 2	○	○	○	○	○
Language 3	○	○	○	○	○
Language 4	○	○	○	○	○
Other	○	○	○	○	○

End of Block: SECTION 1: DEMOGRAPHIC INFORMATION

Start of Block: SECTION 2: EDUCATIONAL BACKGROUND AND TRAINING

SECTION 2 SECTION 2: EDUCATIONAL BACKGROUND AND TRAINING

Q2.1 Please indicate your highest educational level completed.

○ Bachelor's degree

○ Postgraduate or master's degree

○ PhD

X→

Q2.2 In which country did you receive your SLT/SLP training? You can select multiple countries as appropriate.

Q2.3 Did you receive training in any discipline other than SLT/SLP? If you answer yes, please specify any other educational background.

○ Yes ____

○ No

Q2.4 Which of the following options best describes any formal academic or clinical training you have received on **bi/multilingualism (not specific to aphasia)**? You can select more than one response.

	During SLP/SLT studies	After I graduated as SLP/SLT
I have not received any	☐	☐
Independent reading of the relevant literature	☐	☐
Lectures and/or conferences (a few hours) outside the curriculum taken on my own initiative (less than 5 hours)	☐	☐
Just a few classes	☐	☐
One full designated course	☐	☐
More than one full course	☐	☐

Q2.5 Which of the following options best describes any formal academic or clinical training you have received on **bi/multilingual aphasia**? You can select more than one response.

	During SLP/SLT studies	After I graduated as SLP/SLT
I have not received any	☐	☐
Independent reading of the relevant literature	☐	☐

Lectures and/or conferences (a few hours) outside the curriculum taken on my own initiative (less than 5 hours)	▢	▢
Just a few classes	▢	▢
One full designated course	▢	▢
More than one full course	▢	▢

Display This Question:

If Which of the following options best describes any formal academic or clinical training you have r... != I have not received any

Q2.5' Please indicate the content of the **bi/multilingualism AND/OR bi/multilingual aphasia** course(s) you attended (e.g., aphasia assessment, aphasia rehabilitation, psycholinguistics, sociolinguistics, or other)

____

Q2.6 Please use the following scale from "1= Not well at all" to "5= Extremely well" to indicate which value reflects best your SLP/SLT training background.

	1 Not well at all	2 Not so well	3 Somewhat well	4 Very well	5 Extremely well
How well do you consider your training has prepared you to effectively **conduct assessment** on bi/multilingual people with aphasia?	○	○	○	○	○
How well do you consider your training has prepared you to effectively **provide**	○	○	○	○	○

**treatment** for bi/multilingual people with aphasia?					
How well familiarized are you with **theories** of bi/multilingualism?	○	○	○	○	○

Q2.7 Use the scale below to indicate the importance of the following items as possible areas that training programs should emphasize to improve professional preparation to work with bi/multilingual PWA via theoretical courses and/or practical training from "1= Very unimportant" to "5= Very important".

	1 Very unimportant	2 Somewhat unimportant	3 Somewhat important	4 Important	5 Very important
Bi/multilingual language acquisition and learning	○	○	○	○	○
Working with interpreters/translators	○	○	○	○	○
Assessment of cognition in bi/multilingual PWA	○	○	○	○	○
Assessment of premorbid language proficiency in bi/multilingual PWA	○	○	○	○	○
Assessment and treatment of minority/heritage languages in bi/multilingual PWA	○	○	○	○	○
Language assessment tools and procedures for bi/multilingual PWA	○	○	○	○	○
Diverse patterns of language impairment/recovery considering both premorbid and	○	○	○	○	○

postmorbid language abilities					
Identifying the clinical profile of aphasia and other neurogenic communication disorders in bi/multilingual PWA	○	○	○	○	○
Research on bi/multilingual PWA that informs clinical practice in speech-language pathology services	○	○	○	○	○
Treatment for speech and language disorders in bi/multilingual PWA	○	○	○	○	○
Other (please specify)	○	○	○	○	○

Q2.8 On the scale below, choose the number that best represents your interest in participating in continuing education or training opportunities related to bi/multilingual PWA from "1= Not at all interested" to "5= Extremely interested".

	1 Not at all interested	2 Mildly interested	3 Moderately interested	4 Quite interested	5 Extremely interested
Your interest in participating in continuing education or training opportunities related to bi/multilingual PWA	○	○	○	○	○

Q2.9 Is there anything missing in your educational/professional training that would allow you to improve the clinical services you provide to **bi/multilingual people with aphasia**?

◯ Yes

◯ No

Display This Question:

If Is there anything missing in your educational/professional training that would allow you to impro... = Yes

Q2.9' If your answer to the previous question was YES, please use the following scale from 1 to 5 (1= Not important at all; 5= Extremely important) to indicate the importance of the following aspects that, if included in training, would allow you to improve the clinical services you provide to bi/multilingual people with aphasia.

	1 Not important at all	2 Not so important	3 Somewhat important	4 Very important	5 Extremely important
Access to relevant literature	◯	◯	◯	◯	◯
Access to educational programs (courses, workshops, etc.)	◯	◯	◯	◯	◯
Training on how to work with interpreters	◯	◯	◯	◯	◯
Supervised clinical practice with bi/multilingual PWA	◯	◯	◯	◯	◯
Best practice recommendations/guidelines for assessment and treatment of bi/multilingual PWA	◯	◯	◯	◯	◯
Other : please specify	◯	◯	◯	◯	◯

Page Break

SECTION 3 SECTION 3 : CLINICAL SERVICES FOR BI/MULTILINGUAL PEOPLE WITH APHASIA

Q3.1 In standard practice, how often do you work with **people with aphasia** (regardless of whether they are bi/multilingual or monolingual)?

○ Daily

○ Most days of the week

○ Once or twice per week

○ Only a few days per month

○ Other (please specify) ____

Q3.2 What percentage of **people with aphasia** do you see in the clinic per year?

○ 0-20% of my general caseload

○ 21-40% of my general caseload

○ 41-60% of my general caseload

○ 61-80% of my general caseload

○ 81-100% of my general caseload

Q3.3 How often do you work with **bi/multilingual people with aphasia**?

- ○ Daily
- ○ Most days of the week
- ○ Once or twice per week
- ○ Only a few days per month
- ○ Other (please specify) ____

Q3.4 What percentage of **bi/multilingual people with aphasia** do you see in the clinic per year?

- ○ 0-20% of my aphasia caseload
- ○ 21-40% of my aphasia caseload
- ○ 41-60% of my aphasia caseload
- ○ 61-80% of my aphasia caseload
- ○ 81-100% of my aphasia caseload

Q3.5 On the scale below, select the number that indicates how satisfied you are with the resources available to provide speech-language services with bi/multilingual PWA in general from "1= Very dissatisfied" to "5= Very satisfied".

	1 Very dissatisfied	2 Dissatisfied	3 I don't know	4 Satisfied	5 Very satisfied
How satisfied are you with the resources available to **conduct assessment** on	○	○	○	○	○

bi/multilingual PWA? How satisfied are you with the resources available to **provide treatment** for bi/multilingual PWA?	○	○	○	○	○

Q3.6 Do you assess bi/multilingual language background in **people with aphasia** (age of acquisition, proficiency in each language, language use in everyday life, at home, at work, etc.)? If so, do you elaborate your own questions or do you use a multilingual language questionnaire?

☐ YES, I prepare my own set of questions

☐ Yes, I use a multilingual language questionnaire. (Please specify which questionnaire.)

____

☐ No, I do not assess bi/multilingual language background

Q3.7 Do you conduct **language assessments** with bi-/multilingual people with aphasia in their different languages?

○ No, I complete assessments in **only one** language.

○ Yes, I complete assessments in **more than one** language.

Display This Question:

If Do you conduct language assessments with bi-/multilingual people with aphasia in their different... = Yes, I complete assessments in more than one language.

Q3.7a If you complete **assessments** in more than one language, please specify how

- [ ] I do it myself.
- [ ] With the help of a professional interpreter/translator.
- [ ] With the help of relevant others/caregivers.
- [ ] With the help of other clinical staff.

Display This Question:

If Do you conduct language assessments with bi-/multilingual people with aphasia in their different... = Yes, I complete assessments in more than one language.

Q3.7b Please state in detail, the reasons why you decide to conduct **LANGUAGE ASSESSMENT** with bi/multilingual people with aphasia **in more than one language**

____

____

____

____

____

Display This Question:

If Do you conduct language assessments with bi-/multilingual people with aphasia in their different... = No, I complete assessments in only one language.

Q3.7c Please state in detail the reasons why you decide to conduct **LANGUAGE ASSESSMENT** with bi/multilingual people with aphasia **in just one language.**

____

____

____

____

____

Q3.7d If you perceive any barriers to providing clinical services to **bi/multilingual people with aphasia** (e.g., related to your language competence or the availability of resources needed to provide those services), would you refer a bi/multilingual client to another SLT/SLP professional for assessment?

◯ Yes (Please explain why) ____

◯ No (Please explain why) ____

Q3.8 Do you **provide treatment** to bi-/multilingual people with aphasia in their different languages?

◯ No, I provide treatment in **only one** language

◯ Yes, I provide treatment in **more than one** language

Display This Question:

If Do you provide treatment to bi-/multilingual people with aphasia in their different languages? = Yes, I provide treatment in more than one language

Q3.8a If you provide treatment in more than one language, please specify how:

▢ I do it myself

▢ With the help of a professional interpreter/translator

▢ With the help of relevant others/caregivers

▢ With the help of other clinical staff

Display This Question:

If Do you provide treatment to bi-/multilingual people with aphasia in their different languages? = Yes, I provide treatment in more than one language

Q3.8b Please state in detail, the reasons why you decide to provide **LANGUAGE TREATMENT** to bi/multilingual people with aphasia **in more than one language**

____

Display This Question:

If Do you provide treatment to bi-/multilingual people with aphasia in their different languages? = No, I provide treatment in only one language

Q3.8c Please state in detail, the reasons why you decide to provide **LANGUAGE TREATMENT** to bi/multilingual people with aphasia **in just one language**

____

Q3.8d If you perceive any barriers to providing clinical services to bi/multilingual people with aphasia (e.g., related to your language competence or the availability of resources needed to provide those services), would you refer a bi/multilingual client to another SLT/SLP professional for **language treatment**?

- ○ Yes (Please explain why) ____
- ○ No (Please explain why) ____

Q3.9 When you offer language treatment to **bi/multilingual people with aphasia**, what is the rationale that helps you choose the language to target in treatment? Please use the following scale from 1-5 (1 = Not important at all; 5 = Extremely important) to indicate how important each of the statements listed below is in your choice of treatment language.

	1 Not important at all	2 Not so important	3 Somewhat important	4 Very important	5 Extremely important
I provide treatment in my dominant language (being fluent enables	○	○	○	○	○

me to provide better quality treatment).					
I provide treatment in the official language predominantly spoken in the region/country (please specify the language/region).	○	○	○	○	○
I provide treatment in the language that is more relevant for the client (they decide what is most useful for them).	○	○	○	○	○
I provide treatment in the language in which materials are available.	○	○	○	○	○
I provide treatment in the language that has more chances to benefit both the treated and untreated language (according to research or clinical experience).	○	○	○	○	○
I provide treatment in either/any language; choosing a language is not really important.	○	○	○	○	○
I provide treatment in the language(s) in which an	○	○	○	○	○

interpreter is available.					
I provide treatment in the language that is preserved more and has the least impairment postmorbid	○	○	○	○	○
Other (please specify)	○	○	○	○	○

Q3.10 Please answer the following questions on a scale from 1 to 5 to indicate how difficult you find each of these areas when you provide clinical services to **bi/multilingual people with aphasia** (MPWA)? (From 1=Very easy to 5=Very difficult).

	1 Very easy	2 Easy	3 Moderate	4 Difficult	5 Very difficult
How difficult do you find it to **assess** MPWA **in all languages** s/he speaks?	○	○	○	○	○
How difficult do you find it to **assess** MPWA in a language that you are **HIGHLY proficient in**?	○	○	○	○	○
How difficult do you find it to **assess** MPWA in a language that you are **NOT highly proficient** in?	○	○	○	○	○
How difficult do you find it	○	○	○	○	○

to provide **treatment** to MPWA **in all languages** s/he speaks?					
How difficult do you find it to provide **treatment** to MPWA in a language that you are **HIGHLY proficient** in?	○	○	○	○	○
How difficult do you find it to provide **treatment** to MPWA in a language that you are **NOT highly proficient** in?	○	○	○	○	○

Q3.11 In your view, what are the most common challenges SLT/SLPs face when providing services to bi/multilingual people with aphasia? You may select multiple options.

- ▢ Limited or no availability of clinical tools translated to/validated in a bi/multilingual client's language(s)
- ▢ Limited or no linguistic competence to provide appropriate clinical services in a patient's relevant language
- ▢ Limited time to provide clinical services in more than just one language
- ▢ Other (Please specify) ____

Page Break

SECTION 4 SECTION 4 : CLINICAL TOOLS AND THERAPY CONSIDERATION FOR BI/MULTILINGUALS WITH APHASIA

Q4.1 Do you use any version of the aphasia assessment tools listed below to provide clinical services for **bi/multilingual people with aphasia (MPWA)**? This is based on the “List of the 20 Most Commonly Spoken Languages and Corresponding Available Aphasia Tests” published in Ivanova and Hallowell (2013).

	I use this for the diagnostic assessment of MPWA	I use this to assess treatment gains in MPWA	I am familiar with it and it is available in my language, but I opt not to use it	I am familiar with but is not available in my language	I am not aware of it
Aachen Aphasia Test (AAT)	☐	☐	☐	☐	☐
Bilingual Aphasia Test (BAT)	☐	☐	☐	☐	☐
Boston Diagnostic Aphasia Examination (BDAE)	☐	☐	☐	☐	☐
Comprehensive Aphasia Test (CAT)	☐	☐	☐	☐	☐
Psycholinguistic Assessments of Language Processing in Aphasia (PALPA)	☐	☐	☐	☐	☐
Token Test	☐	☐	☐	☐	☐
Western Aphasia Battery (WAB)	☐	☐	☐	☐	☐
OTHER (non listed here, please specify)	☐	☐	☐	☐	☐
OTHER (non listed here, please specify)	☐	☐	☐	☐	☐

OTHER (non listed here, please specify)	☐	☐	☐	☐	☐

Q4.2 Please list any standardized aphasia assessment tools that are currently not available in any of the languages you use to provide clinical services to **bi/multilingual people with aphasia**. Also, please state the language in which each tool would be needed.

____

____

____

____

____

Q4.3 There is evidence that some forms of language/aphasia treatment may benefit treated and untreated languages. Do you use any of these treatments to promote cross-language generalization?

☐ Semantics-based treatment

☐ Cognate-based treatment

☐ Phonology-based treatment

☐ None of the above

☐ Other treatment (please specify)

____

☐ I was not aware that certain types of treatment promote cross-language generalization

Q4.4 Please state any other concerns that you may have regarding the provision of clinical services (assessment and treatment) to **bi/multilingual people with aphasia** that have not been addressed in this survey.

____

End of Block: SECTION 2: EDUCATIONAL BACKGROUND AND TRAINING

Start of Block: Responder ID

comments Please provide any comments or feedback (Optional).

____

THANK YOU
Thank you for your participation in this survey!

Please make sure you keep the following unique Responder ID in your message and provide it in any correspondence you may need to keep with the developers of this survey. This will enable us to locate and retrieve your data.

This is your unique Responder ID:${e://Field/Random%20ID}

Please press the submit button to submit your responses.

End of Block: Responder ID
